# Interaction between Rho GTPases and 14-3-3 Proteins

**DOI:** 10.3390/ijms18102148

**Published:** 2017-10-15

**Authors:** Daniel Brandwein, Zhixiang Wang

**Affiliations:** Department of Medical Genetics and Signal Transduction Research Group, Faculty of Medicine and Dentistry, University of Alberta, Edmonton, AB T6G 2H7, Canada; brandwei@ualberta.ca

**Keywords:** Rho GTPases, 14-3-3 protein, Rac1, interaction, guanine nucleotide exchange factors (GEFs), GTPase-activating proteins (GAPs), phosphorylation

## Abstract

The Rho GTPase family accounts for as many as 20 members. Among them, the archetypes RhoA, Rac1, and Cdc42 have been the most well-characterized. Like all members of the small GTPases superfamily, Rho proteins act as molecular switches to control cellular processes by cycling between active, GTP-bound and inactive, GDP-bound states. The 14-3-3 family proteins comprise seven isoforms. They exist as dimers (homo- or hetero-dimer) in cells. They function by binding to Ser/Thr phosphorylated intracellular proteins, which alters the conformation, activity, and subcellular localization of their binding partners. Both 14-3-3 proteins and Rho GTPases regulate cell cytoskeleton remodeling and cell migration, which suggests a possible interaction between the signaling pathways regulated by these two groups of proteins. Indeed, more and more emerging evidence indicates the mutual regulation of these two signaling pathways. There have been many documented reviews of 14-3-3 protein and Rac1 separately, but there is no review regarding the interaction and mutual regulation of these two groups of proteins. Thus, in this article we thoroughly review all the reported interactions between the signaling pathways regulated by 14-3-3 proteins and Rho GTPases (mostly Rac1).

## 1. Introduction

The 14-3-3 family proteins function by binding to Ser/Thr phosphorylated intracellular proteins, which alters the conformation, activity, and subcellular localization of their binding partners [1,2]. The 14-3-3 proteins interact with a wide spectrum of proteins involved in cell signaling, transcription regulation, cytoskeleton remolding, DNA repair, and apoptosis. Therefore, 14-3-3 proteins regulate diverse cell functions including cell cycle, cell development, cell proliferation and cell motility. Recently, a growing number of proteins involved in actin remodeling have been identified as 14-3-3 binding partners [1].

The Rho GTPase family mediate a plethora of cellular processes including regulation of cell size, proliferation, apoptosis/survival, cytoarchitecture, cell polarity, cell adhesion, cell motility and membrane trafficking [3]. The The GTPase family accounts for as many as 20 members. Among them, the archetypes RhoA, Rac1, and Cdc42 have been the most well-characterized [4,5]. Like all members of the small GTPases superfamily, Rho proteins act as molecular switches to control cellular processes by cycling between active, GTP-bound and inactive, GDP-bound states. A major function of Rho proteins is controlling cell cytoskeleton remodeling and cell migration. It has become evident, however, that a simple GTPase cycle cannot solely explain the variety of functions and signaling initiated by Rho proteins. Recent findings have suggested that additional regulatory mechanisms such as post-transcriptional regulation by microRNAs [6], ubiquitination [7], palmitoylation [8], and phosphorylation [9] might contribute further to the tight regulation of Rho GTPases.

Both 14-3-3 proteins and Rho GTPases regulate cell cytoskeleton remodeling and cell migration, which suggests a possible interaction between the signaling pathways regulated by these two groups of proteins. Indeed, more and more emerging evidence indicates the mutual regulation of these two signaling pathways. There have been many documented reviews of 14-3-3 protein and Rac1 separately, but there is no review regarding the interaction and mutual regulation of these two groups of proteins. Thus, in this article we thoroughly review all the reported interactions between the signaling pathways regulated by 14-3-3 proteins and Rho GTPases.

## 2. The 14-3-3 Proteins

### 2.1. Structure and Function of 14-3-3 Proteins

The 14-3-3 proteins, comprising seven isoforms, is a family of evolutionarily conserved, widely expressed homodimeric and heterodimeric acidic proteins that bind to a vast number of intracellular proteins in normal and cancer cells, often by interacting at phosphoserine and phosphothreonine residues [10,11]. The 14-3-3 protein isoforms bind to two phosphorylation-dependent high affinity binding motifs: RSXpSXP (mode I) and RXY/FXpSXP (mode II) [12,13]. In addition to the two binding motifs, 14-3-3 proteins exhibit binding to the extreme C-terminus (pSX1–2–COOH) of numerous proteins, lately defined as mode III [14,15,16]. The 14-3-3 proteins are involved in many dynamic cellular processes, such as mitogenesis, cell cycle control, DNA damage checkpoints, and apoptosis [16,17,18]. The 14-3-3 proteins have been shown to be an integral part in signal transduction pathways and cancer progression; however, the mechanisms have yet to be elucidated [1].

In mammalian cells, the 14-3-3 protein has seven isoforms (β, ε, γ, η, σ, τ, and ζ) encoded by separate genes [16,18,19,20,21]. There are many different 14-3-3 isoforms, yet the protein-binding partners interact on the same sites [22]. The various isoforms provide unique recognition motifs for specific ligands. Most 14-3-3 isoforms form heterodimers in vitro and also form homodimers in vivo, except for 14-3-3ε and ζ, which can form heterodimers in vivo [23].

### 2.2. The 14-3-3 Proteins and Cancer

Global down-regulation of 14-3-3 expression causes tumor suppression, while overexpression of 14-3-3 proteins is often seen in many cancerous phenotypes [24,25,26,27,28,29]. The 14-3-3ζ, β and γ isoforms have been shown to produce oncogenic effects [27,30] specifically, *β, γ, ε, ζ* and *θ* 14-3-3 gene expressions have been shown to be higher in lung cancer [31]. The 14-3-3τ isoform binds to the cyclin-dependent kinase inhibitor p21Waf1/Cip1 and induces ubiquitin-independent proteasomal degradation of p21, promoting cell growth [32]. Overexpression of 14-3-3τ is frequently observed in human breast cancer cells and is associated with lower patient survival, possibly by increasing invasion and metastasis by inhibiting RhoGDIα [33]. However, there has yet to be a direct link between 14-3-3τ overexpression in breast cancer and breast cancer metastasis. Of all the 14-3-3 isoforms, 14-3-3σ and ζ have been most directly linked to cancer. The 14-3-3σ and ζ isoforms produce opposite effects in mammary epithelial cells [34]. The 14-3-3σ isoform is shown to have tumor suppressor effects by inducing cell cycle arrest at the G2-M transition [35]. The 14-3-3σ isoform expression is down-regulated in bladder [36], prostate [37], and ovarian cancers [38]. In contrast, increased expression of 14-3-3ζ has been linked to enhanced tumor growth and inhibition of 14-3-3ζ has been shown to be a targeted therapeutic strategy in the treatment of prostate cancer [39,40].

## 3. Rho GTPases and Rac1

### 3.1. Rho GTPases

Rho GTPases are monomeric, small GTP-binding proteins belonging to the Ras superfamily. Within the Rho GTPases family, RhoA, Rac1, and Cdc42 have been most extensively characterized [41]. Rho GTPases play pivotal roles in the regulation of cell size, proliferation, apoptosis, cell polarity, cell adhesion, cell motility and membrane trafficking [2,3]. Like all other small GTP-binding proteins, the regulatory cycle of Rho GTPases is exerted by three distinct families of proteins: guanine nucleotide exchange factors (GEFs) activate Rho GTPases by promoting the exchange of GDP by GTP. GTPase-activating proteins (GAPs) negatively regulate Rho GTPases by stimulating its intrinsic GTPase activity leading to an inactive GDP-bound state. The guanine nucleotide dissociation inhibitors (GDIs) inhibit the dissociation of GDP from Rho GTPases and prevent the binding of GDP-Rho GTPases to cell membranes. Rho GEFs, GAPs, and GDIs thus have been established as the mainstream regulators of Rho GTPases [4]. The GTPase cycle is essential for Rho GTPase biological functions, leading to interaction with downstream effectors [5,6].

### 3.2. Rac1 and Its Regulation

The Rac subfamily of Rho GTPases includes Rac1 (and its splice variant Rac1b), Rac2 and Rac3 and share high sequence similarity (80%) [42,43]. Rac1 is ubiquitously expressed, Rac2 is expressed in hematopoietic cells [44,45] and Rac3 mRNA is expressed in the brain [46,47,48]. Rho GTPases are best known for their role in regulating the cytoskeleton and regulating gene expression.

Like all the other Rho GTPases, the regulatory cycle of Rac1 is exerted by three distinct families of proteins: the activator GEFs, and two families of suppressors GAPs and GDIs. The cycling of Rac1 between the GTP-bound and GDP-bound states might be required for effective signal flow to elicit downstream biological functions [49,50]. Prenylation also plays a role in the regulation of Rac1 by targeting Rac1 to the plasma membrane and facilitating Rac1 interaction with GEFs [51]. Recent findings suggest that additional regulatory mechanisms such as post-transcriptional regulation by microRNAs [6], ubiquitination [7], palmitoylation [8], and phosphorylation [9] might further contribute to the tight regulation of Rho GTPases. RhoA was the first Rho protein shown to be phosphorylated [52,53,54,55]. Subsequently, the other members of the Rho family, including Cdc42, RhoE, and Rac1 have been shown to be regulated by serine or tyrosine phosphorylation [56,57,58,59].

### 3.3. Phosphorylation of Rac1

**Figure 1:**
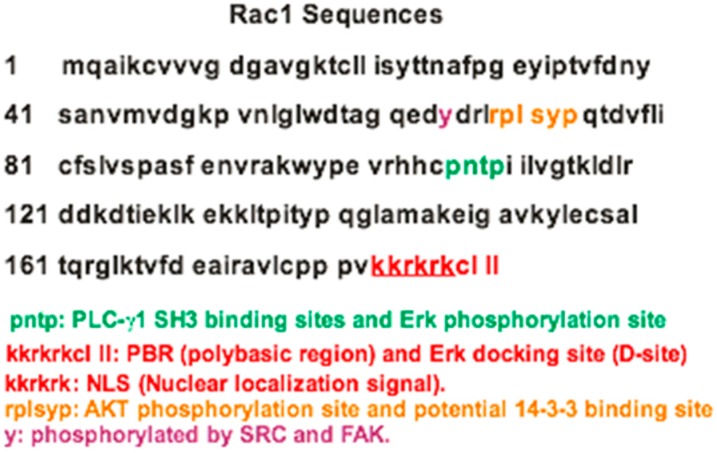
Rac1 sequences, motifs and phosphorylation sites.

Rac1 is reported to be phosphorylated on S71 by Akt [57] (Figure 1). This phosphorylation of Rac1 inhibits its GTP binding activity without any significant change in GTPase activity. Both the GTP-binding and GTPase activities of mutant Rac1 S71A are abolished regardless of the activity of Akt [57]. Moreover, Rac1 may be phosphorylated at Y64 by FAK and SRC kinases (Figure 1). In addition, Y64 phosphorylation targets Rac1 to focal adhesions. Rac1-Y64F displayed increased GTP-binding, increased association with βPIX, and reduced binding to RhoGDI as compared with wild type Rac1. Rac1-Y64D had less binding to PAK than Rac1-WT or Rac1-64F. In vitro assays demonstrated that Y64 in Rac1 is a target for FAK and Src [56]. ERK phosphorylates T108 of Rac1 in response to EGF stimulation [60]. This phosphorylation alters Rac1 activity and subcellular localization and affects Rac1 function in mediating cell migration. The subcellular localization of Rac1 is also regulated by its C-terminal polybasic region (PBR) (Figure 1). It was shown that the PBR of Rac1 has a functional nuclear localization signal (NLS) sequence, which acts as an NLS for protein complexes containing Rac1 [61] (Figure 1). Rac1 is phosphorylated at multiple sites. However, the function and the significance of these phosphorylations are far from clear.

## 4. Interaction between 14-3-3 Proteins and Rho Proteins

**Figure 2:**
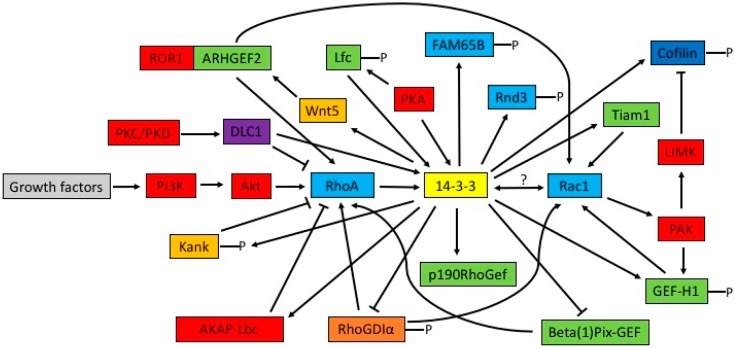
Reported interactions between 14-3-3 proteins and Rho GTPases. The 14-3-3 proteins interact indirectly or directly with many Rho regulators, eventually affecting multiple functions of the Rho GTPases including cytoskeletal remodeling and cell migration. Arrows represent activation and T-bars represent inhibition.

Accumulating evidence suggests that the interaction between the signaling pathways by 14-3-3 proteins and Rho proteins (Figure 2) play important roles in the regulation of cell cytoskeleton remodeling and cell migration.

### 4.1. Regulation of Rho GTPases by 14-3-3 Proteins

Most findings support the role of 14-3-3 proteins regulating Rho GTPases. Many researches indicate that 14-3-3 proteins regulate RhoA through interactions with Rho GEFs and Rho GAPs. Through a yeast two-hybrid screen, 14-3-3η and 14-3-3ε were identified as binding partners of p190RhoGEF. Interactions between p190RhoGEF and 14-3-3η were also confirmed biochemically. Deletion of the 14-3-3 binding site in p190RhoGEF abolishes their interactions in vitro as well as the ability of 14-3-3η to alter the cytoplasmic aggregation of p190RhoGEF in cotransfected cells. The findings suggest a potential role for 14-3-3 in modulating p190RhoGEF activity [62]. Using tandem proteomic and biochemical approaches, the authors identify a phospho-dependent 14-3-3 binding site on the A kinase anchoring protein (AKAP-Lbc). AKAP-Lbc is a GEF for the Rho GTPase. The 14-3-3 protein binding to AKAP-Lbc, induced by protein kinase A (PKA), suppresses Rho activation in vivo [63]. The 14-3-3β isoform negatively affects the GEF activity of dimeric β1Pix only, which suggests a role of 14-3-3β in modulating β1Pix GEF activity [64]. The human *Kank1* gene (KANK1) was found as a candidate tumor suppressor gene for renal cell carcinoma [7]. Kank negatively regulates the formation of actin stress fibers and cell migration through the inhibition of RhoA activity, which is controlled by the binding of KANK1 to 14-3-3 in PI3K-Akt signaling [65]. Deleted in liver cancer 1 (DLC1) is a Rho GAP that is downregulated in various tumor types. In vitro, DLC1 specifically inactivates the small GTPases RhoA, RhoB and RhoC through its GAP domain and this appears to contribute to its tumor suppressor function in vivo. Molecular mechanisms that control DLC1 activity have not so far been investigated. The authors show that phorbol-ester-induced activation of protein kinase C and protein kinase D stimulates association of DLC1 with the phosphoserine/phosphothreonine-binding 14-3-3 adaptor proteins via recognition motifs that involve Ser327 and Ser431. Association with 14-3-3 proteins inhibits DLC1 GAP activity and facilitates signaling by active Rho [66]. The 14-3-3 protein interacts directly and in a phosphorylation-dependent manner with Lfc, which suppresses the exchange activity of wild-type Lfc on RhoA [67].

Studies also support the role of 14-3-3 in the regulation of Rac1. EGF induces biphasic Rac1 activation in the process of cell migration, and UTKO1, a cell migration inhibitor, inhibits the second EGF-induced wave of Rac1 activation but not the first wave. The function of UTKO1 is to abrogate the binding of 14-3-3ζ to Tiam1 that is responsible for the second wave of Rac1 activation, suggesting that the interaction of 14-3-3ζ with Tiam1 is involved in the activation of Rac1 [68]. Previous studies have said that 14-3-3ζ also could mediate the integrin-induced activation of Cdc42 and Rac1 [69]. For instance, Tiam1 is recruited to β1-integrin complexes by 14-3-3ζ, where it mediates integrin-induced Rac1 activation and motility [70]. Expression of wild-type 14-3-3ζ significantly enhanced Rac activity in PC3 cells, which enhances prostate cancer cell–matrix interactions, motility and transendothelial migration [39]. In contrast, silencing 14-3-3ζ inhibits Rac1 activation and decreases lamellipodia formation [39,71,72]. The activation of human T-cells through the T-cell receptor (TCR) eventually leads to the binding and interaction between Tiam1 and 14-3-3 protein, thereby activating Rac1 [73]. The complex of Tiam1 and 14-3-3 protein subsequently leads to downstream cytoskeleton remodeling, cell adhesion and cell migration. One study shows that the loss of Par3 promotes 14-3-3ζ binding to Tiam1, which triggers the high levels of Rac-GTP [74]. Activated Rac1 then triggers JAK-STAT pathway activation, resulting in lung adenocarcinoma tumor growth, cell proliferation, angiogenesis and metastasis. The 14-3-3 protein binds to phosphorylated Rnd3, an atypical constitutively GTP-bound Rho protein, and inhibits Rnd3-induced cell rounding by translocating it from the plasma membrane to the cytosol [75].

Lfc is a GEF for Rho that demonstrates an unusual ability to associate with microtubules. It was reported that Lfc is phosphorylated in an AKAP-dependent manner by PKA. It was shown that a specific isoform, 14-3-3τ, promotes breast cancer metastasis, in part through binding to and inhibition of RhoGDIα, a negative regulator of Rho GTPases and a metastasis suppressor. The 14-3-3τ isoform binds Ser174-phosphorylated RhoGDIα and blocks its association with Rho GTPases, thereby promoting epidermal growth factor (EGF)-induced RhoA, Rac1, and Cdc42 activation [33]. Rgf1p nuclear accumulation during replication arrest depends on the 14-3-3 chaperone Rad24p and the DNA replication checkpoint kinase Cds1p. Both proteins control the nuclear accumulation of Rgf1p by inhibition of its nuclear export, which is part of the mechanism by which Cds1p and Rad24p promote survival in the presence of chronic replication stress [76]. Rad24, a yeast 14-3-3 protein regulates the availability of Cdc42 GEF Gef1 (a homologue of mammalian Cdc42 GEF DNMBP/TUBA), which spatially controls Cdc42 GTPase activity and stimulates cell polarization. Loss of the Rad24-Gef1 interaction increases Gef1 protein localization and Cdc42 activation at the cell tips and reduces the anticorrelation of active Cdc42 oscillations [77]. It was also shown that 14-3-3 proteins bind to phosphorylated FAM65B, a newly identified RhoA inhibitor, which stabilize FAM65B [78]. The 14-3-3ζ isoform plays a crucial role in regulating cytoskeletal structures, ECM homeostasis, and TGF-β1-induced contraction in TM cells by acting through the RhoA signaling pathway [79]. A recent study reveals that 14-3-3ζ plays a critical role in Wnt5a induced recruitment of ARHGEF2 to ROR1, activation of ARHGEF2 and the subsequent activation of RhoA and Rac1 in CLL cells [80].

### 4.2. 14-3-3 Downstream of Rho GTPases

Some early data also suggest that 14-3-3 protein could act downstream of Rho GTPases. It has been reported that 14-3-3 acts downstream of RacE, associates with myosin II heavy chain, and is needed to promote myosin II bipolar thick filament remodeling [81]. Rho GTPases act through p21-activated kinase-1 (PAK1) and Rho kinase to inhibit cofilin activity via the LIM kinase (LIMK)-mediated phosphorylation of cofilin on Ser3. It has been shown that 14-3-3ζ binds to phosphocofilin. The expression of 14-3-3ζ increases phosphocofilin levels, and the coexpression of 14-3-3ζ with LIMK further elevates phosphocofilin levels and potentiates LIMK-dependent effects on the actin cytoskeleton. This potentiation of cofilin action appears to be a result of the protection of phosphocofilin from phosphatase-mediated dephosphorylation by bound 14-3-3ζ. Taken together, these results suggest that Rho GTPases regulate the binding between 14-3-3ζ and phosphocofilin through PAK1, which regulates cellular actin structures through the maintenance of phosphocofilin levels [82].

### 4.3. Mutual Regulation of Rho and 14-3-3 Proteins

It is interesting to notice the mutual regulation of Rho and 14-3-3 proteins through GEF-H1 and PAK1. PAKs are serine/threonine kinases whose activity is regulated by the binding of activated Rac or Cdc42 [83]. In an independent biochemical screen for novel substrates of PAK1, GEF-H1 was identified as a phosphorylation target of PAK1. The phosphorylation of GEF-H1 at Ser885 by PAK1 regulates the docking of 14-3-3 to GEF-H1 and its recruitment to microtubules. These observations suggest that PAK and 14-3-3 are involved in regulation of GEF-H1 activity, which may act to coordinate Rac/Cdc42- and Rho-dependent signaling pathways [84].

The presumed interactions in the articles mentioned above between 14-3-3 and Rac1 are indirect. When this mini-review was written, there was yet to be data suggesting a direct interaction between 14-3-3 protein isoforms influencing Rac1 function, stability or subcellular localization.

## 5. Future Direction: A Direct Interaction between Rac1 and 14-3-3 Protein?

So far, all the data regarding the interaction between Rho GTPases and 14-3-3 proteins are indirect. Either 14-3-3 proteins regulate Rho GTPases through the interaction with Rho regulators or Rho GTPases control 14-3-3 proteins through PAK1. However, it is possible that Rho GTPases, particularly Rac1, could interact with 14-3-3 protein directly. Rac1 is phosphorylated on S71 by Akt, leading to inhibition of GTP binding, but not Rac1 GTPase activity [57]. Examination of the Rac1 amino acid sequence shows that the motif RPLpSYP is close to the consensus binding motif for 14-3-3 protein [85,86], which suggests that S71 phosphorylation could regulate Rac1 interaction with 14-3-3 proteins. The interaction between 14-3-3 protein and Rac1 by S71 phosphorylation regulates subcellular localization, stability and activity functions in the cell. The 14-3-3 protein may also assist in the localization of activated Rac1 to membrane ruffles [87]. Interestingly, a recent study indicated that Rnd3 directly interacts with 14-3-3 protein through its C-terminal site, consisting of both the Cys241-farnesyl moiety and a Rho-associated coiled coil containing protein kinase (ROCK)-dependent Ser240 phosphorylation site [75]. However, as Rac1 is geranylgeranylated, rather than farnesylated at the C-terminal, and S71 is far away from the C-terminal, the mechanism underlying its interaction with 14-3-3 protein could be different.
